# Book Reviews

**Published:** 1899-12

**Authors:** 


### Book Reviews.

A Treatise on Surgery. By American authors. Edited by Roswell Park, M. D., Professor of Surgery in the University of Buffalo, N. Y. New condensed edition in one royal octavo volume of 1262 pages, with 625 engravings and thirty-seven full-page plates in colors and monochrome. Philadelphia and New York: Lea Brothers & Co.

When the first edition of this work appeared, now some three years gone, it was in two volumes, of which the Journal entered into somewhat extended consideration. [December, 1896, p. 385.] At present we shall content ourselves with general rather than special observations on this edition.

In the first place we note the condensation of the work into one volume. On some accounts a one volume treatise is more satisfactory; it can be readily consulted without loss of time; it is cheaper in price; and, moreover, it is quite as apt to be succinct without detriment to its value as a text-book. As against this a single volume is likely to be somewhat clumsy or weighty to handle—this being the case in the present instance. However, all things considered, we think a single volume treatise is in the main to be preferred, and we have no doubt in this particular case the change will contribute to increase the popularity of the work.

Next, it may be observed that there are two editions of the work extant, a somewhat novel circumstance, one, however, which will enable a designing purchaser to choose for himself as between the one and the two volume editions. In this relation it may be in place to note that the reduction in price of the one volume edition has been proportionately much greater than the reduction of the material. There are many teachers who will naturally prefer the two volume edition, and probably a good many practitioners who will be like-minded. On the other hand, a large number of students and surgeons will find the one volume adequate.

It is probable that editor and publisher have been greatly surprised at the success the work achieved so soon after its publication, hence the issue of this condensed style, without material reduction of the matter it contains.

In our former notice we made special reference to the section on surgical pathology, of which the editor is the author. This section, at first, contained so much that was new, it has hardly yet been overtaken by the advance of science. However, the author has made some revisions of the text to keep the several topics dealt with abreast of his own studies and investigations. He is particularly happy in his treatment of the subjects embraced in this section, especially in the distinction made between hyperemia and inflammation, the insistence upon the practical importance of bacteriology, the development of autointoxications, and his teaching as to the surgical pathology of the blood. It is demonstrated in this section that Professor Park is a painstaking worker, who appreciates the importance of combining laboratory research and clinical study, insisting upon exactitude in both as far as it is possible to do so.

The contributions by a large staff of eminent surgeons are most creditable in character and indicate the progressive American spirit. Nowhere is medicine more advanced than in this country, and the fact is amply attested by the contributions to this treatise. We made previously detailed comment as to their several parts, which we need not repeat now as what we said holds equally good today and with even greater cogency. It may be well, however, to recall their names for the benefit of any who may not have seen the first edition. They are: William T. Belfield, Arthur Dean Bevan, Clarence J. Blake, Edward H. Bradford, Charles Stedman Bull, Herbert L. Burwell, D. Bryson Delavan, Frederic S. Dennis, James H. Etheridge (now deceased), Duncan Eve, John A. Fordyce, Frederic H. Gerrish, Arpad G. Gersler, William A. Hardaway, Hobart Amory Hare, James M. Holloway, Charles B. Kelsey, Robert W. Lovett, Rudolph Matas, Henry H. Mudd, Charles B. Nancrede, Roswell Park (the editor), Charles B. Parker, John Parmenter, Joseph Ransohoff, Maurice H. Richardson, Chauncey Pelton Smith, and Edmond Souchon. It would be difficult to form a second group, equal in number, of eminent American surgeons without including any of these. Their work makes the treatise distinctively American in character—an interesting fact. While science is for all the world and not for any geographic division thereof, it is yet pleasant to contemplate that here is one of the best treatises extant on surgery, written wholly by American authors, containing much material that is really even in advance of its period of issue, as well as preserving all the accumulation of the past that has stood the test of time.

A word should be said as to the illustrations. These have commanded the special attention of the editor, most of which have been made expressly for this treatise. Colors have been frequently used, wherever, indeed, they could be made available to bring out the subject of a plate more potently. The camera has been most skilfully employed in a large number of original cases, and wood engravings, etchings, and drawings unite to give variety and accuracy to the illustrations.

Finally, the treatise is to be commended to every student of surgery —undergraduate or practitioner—who would wish to obtain the best, and to study it attentively with a view to accepting it as a guide.

THE MEDICAL COMPLICATIONS, ACCIDENTS AND SEQUELÆ OF TYPHOID OR ENTERIC FEVER. By HOBART AMORY HARE, M. D., B. Sc., Professor of Therapeutics in the Jefferson Medical College of Philadelphia; Physician to the Jefferson Medical College Hospital; Laureate of the Medical Society of London, of the Academie Royale de Médecine de Belgique, etc. With a special chapter on the Mental disturbances following typhoid fever. By F. X. Dercum, M. D., Clinical Professor of Diseases of the Nervous System in the Jefferson Medical College. Octavo, 267 pages, twenty-one engravings and two full-page plates. Philadelphia and New York: Lea Brothers & Co. 1899.

This monograph deals with the complications of a disease of world-wide prevalence. Within the recollection of the writer, the profession has changed front in the management of the malady two or three times. First, it was depletion, next stimulation, and now it is reduction of temperature and cautious nutrition. This statement, of course, applies to classic typhoid. There are all degrees of variation admissible in the management of aberrant forms of the disease such as this treatise deals with. It is these irregular manifestations that tax the knowledge of the diagnostician and resource of the therapeutist, often putting them to the severest test.

On this account such a book as Hare's will be welcomed by the general practitioner of medicine, the doctor residing in the small city, village or hamlet, because he can draw from it aid in those obscure varieties of the malady that he is accustomed to meet and for which he needs the promptest and best advice. Without doubt one of the most essential questions relative to the consideration of typhoid fever is diagnosis, and in its obscure or aberrant forms this often becomes most difficult, even to the experienced clinician. This is most true when typhoid fever and malaria are interblended, as they so frequently become nowadays, especially during military campaigns. This is most strikingly set forth in the recent paper of Dr. Victor C. Vaughan, editorial comment upon which we made in the edition of the JOURNAL for June, 1899.

The present book, with which we have especially to deal at this time, is made up of seven chapters, embracing the following titles: (1) General consideration; (2) varieties of onset; (3) The aberrant symptoms, status or complications of the well-developed stage of the disease; (4) Complications of the period of convalescence; (5) The conditions which ape typhoid fever; (6) duration and immunity to second attack; and (7) the mental complications. From these headings it will be observed that almost every complication or form of aberrance that the disease may take on is herein dealt with, and, it is needless to add, in the most intelligent, clear and scientific way. The monograph of Keen on the surgical complications and sequelæ of typhoid fever, and this one of Hare's dealing with its medical complications, constitute the most important contributions to the recent literature of a disease with which every American physician should become familiar.

---

PUBLIC HEALTH REPORTS. Issued by the Supervising Surgeon-General Marine Hospital Service, under the national quarantine act of April 29, 1878, and the act granting additional quarantine powers and imposing additional duties upon the Marine Hospital Service, approved February 15, 1893. Volume XIII. Nos. 1 to 52. Washington: Government Printing Office. 1899.

ANNUAL REPORT OF THE SUPERVISING SURGEON-GENERAL OF THE MARINE HOSPITAL SERVICE OF THE UNITED STATES, for the fiscal year 1898, Centennial Year. Octavo, pp. 855. Washington: Government Printing Office. 1899.

These two books furnish substantial evidence of the progress making in the fields of public health and preventive medicine. The first named—Public Health Reports— is a handsome volume, of over 1,500 pages, bound in red cloth, containing the regular weekly reports of the service that have been punctually received by the JOURNAL. It is an excellent plan, however, for the department to issue them in book form at the end of the year, as it affords a convenient reference volume for all interested in vital statistics, or any other question pertaining to the public health. Besides there are in it many valuable reports and papers relating to infectious and epidemic diseases, such as cholera, yellow fever, plague, smallpox and the like.

The second book, of which the title appears above, is an elaborate report by Dr. Walter Wyman, the supervising Surgeon-General of the Marine Hospital Service, for the fiscal year ending June 30, 1898, which is also the centennial year of the service. It is accompanied by the usual documents relating to the conduct of the service all over the world and contains much of special interest to public health officers.

It was the intention of Dr. Wyman, as he declares in his report, to have included at this time a history of the development of the service, together with a full description of its present legal status, its functions and personnel; but the war with Spain and the added work on account of the yellow fever outbreak in the south, superseded all other demands upon his time, and he has, in consequence, been obliged to defer his historical summary until his next annual report. In this one, however, there is much interesting material that deserves examination at the hands of all engaged in the public health service.

---

THE PHYSICIAN'S VISITING LIST (Lindsay & Blakiston's) FOR 1900. Forty-ninth year of its publication. Philadelphia: P. Blakiston's Son & Co. 1900.

This old favorite has again been placed on the market with its usual promptitude. It has stood the test of time and contains everything needful in the way of a pocket memorandum of daily work for the busy physician. It is furnished in several styles at different prices—a full description will be found on page xvi. of our advertising columns, *q. v.* It is simple in make up, compact and convenient.

It is for sale by all booksellers, or it will be sent by the publishers, 1012 Walnut Street, Philadelphia, postage prepaid, on receipt of price.

---

THE MEDICAL NEWS VISITING LIST FOR 1900. Weekly (dated, for 30 patients); monthly (undated, for 120 patients per month); perpetual (undated, for 30 patients weekly per year); and perpetual (undated, for 60 patients weekly per year). The first three styles contain 32 pages of data and 160 pages of blanks. The 60-patient perpetual consists of 256 pages of blanks. Each style in one wallet-shaped book, with pocket, pencil and rubber. Seal grain leather, $1.25. Thumb-letter index, 25 cents extra. Philadelphia and New York: Lea Brothers and Co.

Since a visiting list of some sort has become indispensable the only question for a physician to determine is, which one of the several good ones on the market shall be purchased? In making a selection for the first year of the new century, the *Medical News Visiting List* will be found in the front rank and on the first shelf.

Its blank pages are arranged to classify and record memoranda and engagements of every description occurring in the practice of the physician, surgeon or obstetrician.

The book opens with 32 pages of printed data of the most useful sort, including an alphabetical table of diseases with approved remedies, a table of doses, sections on examination of urine, artificial respiration, incompatibles, poisons and antidotes, a diagnostic table of eruptive fevers, and a full-page plate, showing at a glance the incisions for ligation of the various arteries, a useful guide in such emergencies. It is issued in four styles, adapted to any system of records and any method of keeping professional accounts. It is printed on fine, tough paper, suitable for pen or pencil, and durably and handsomely bound in the size of a wallet for the pocket. When desired a ready reference thumb-letter index is furnished, which is an economiser of time. Taken as a whole, it is one of the best, and no mistake can be made in purchasing it.

